# Live spatial validation of plant-microbe interactions in the rhizosphere with light-sheet fluorescence microscopy

**DOI:** 10.3389/fpls.2026.1953583

**Published:** 2026-10-05

**Authors:** Xingshui Dong, Qilu Cheng, Junwei Ma, Feng Wang, Hui Lin

**Affiliations:** State Key Laboratory for Quality and Safety of Agro-Products, Zhejiang Provincial Key Laboratory of Agricultural Microbiomics, Institute of Environment, Resource, Soil and Fertilizer, Zhejiang Academy of Agricultural Sciences, Hangzhou, China

**Keywords:** correlative microscopy, light-sheet fluorescence microscopy, live imaging, plant-microbe interactions, rhizosphere, spatial validation, transparent soil

## Abstract

Plant-microbe interactions in the rhizosphere are spatial events, yet mechanisms are often inferred from bulk samples, endpoint images, or community profiles averaged across heterogeneous root zones. Rather than surveying LSFM hardware or rhizosphere imaging broadly, this review evaluates light-sheet fluorescence microscopy (LSFM) as a platform for claim-level live spatial validation. Evidence is separated into four levels: A, direct LSFM evidence; B, an LSFM-compatible sample or device demonstrated with another modality; C, a correlative or validation modality; and D, a proposed future application. Within the verified full-text evidence set, direct evidence is concentrated in whole-rhizosphere tracking of Bacillus populations, plant reporter responses during early root-fungus interactions, and rhizobial attachment and infection of root hairs. Many arbuscular mycorrhizal and biocontrol applications remain prospective. We compare instrument and sample choices across field of view, numerical aperture, voxel sampling, time interval, spectral channels, and matrix realism; define root-centric metrics for arrival, residence, occupancy, growth, and persistence; and distinguish simultaneous, sequential *in situ*, and destructive endpoint validation. LSFM can identify where and when a candidate interaction occurs, but identity, activity, perturbation, and functional assays are still required before mechanism is assigned. A minimum reporting checklist is proposed to connect optical settings, environmental context, hierarchical sampling, registration error, and open analysis. The strongest role of LSFM in rhizosphere research is therefore targeted and evidence-bounded: resolving a live spatial prediction and directing independent tests of what the interaction does and why.

## 1 Introduction

The rhizosphere is not a uniform compartment surrounding the root. It is a moving interface in which axial root zones, radial distance from the root surface, pore connectivity, moisture, oxygen, exudate chemistry, microbial motility, and plant developmental state vary over micrometres to millimetres and minutes to days. Plant-microbe interactions therefore occur as local encounters before they appear as changes in community composition, metabolite profiles, plant health, disease symptoms, or nutrient fluxes (Berendsen et al., 2012). This spatial nature has long been recognized in rhizosphere biology, but it is still difficult to measure directly because the relevant events are small, dynamic, and embedded in opaque or semi-opaque growth environments (Downie et al., 2015; Kuzyakov and Razavi, 2019; Oburger and Schmidt, 2016).

Bulk sampling, sequencing, qPCR, metabolomics, and isotope tracing have transformed the field, yet they often separate biological process from physical location. A root washing sample may identify a responsive taxon, a destructive harvest may detect a nutrient-cycling gene, and a rhizosphere extract may reveal exudate chemistry, but these measurements rarely show whether the organism, signal, or gene occupied the active root zone before the response occurred. The Spatially Explicit, Resolved, and Situationally contextualized sampling (SERS) framework formalized this requirement as distance, time, and context (DTC) (Etesami, 2026). Here, SERS refers to that sampling framework, not surface-enhanced Raman spectroscopy.

Light-sheet fluorescence microscopy (LSFM) is relevant to this gap because planar illumination can reduce out-of-focus excitation and support repeated volumetric sampling when the sheet geometry, exposure, view number, reporter brightness, and experiment duration are appropriately matched. In plant research, LSFM has supported long-term imaging of root growth, cellular dynamics, root-hair behaviour, and organ development (Maizel et al., 2011; Sena et al., 2011; Yang et al., 2013; von Wangenheim et al., 2017; Ovecka et al., 2022). In live plant-environment and rhizosphere systems, direct LSFM evidence is narrower and includes root-associated bacterial populations and early plant responses during microbial interaction; adjacent transparent-soil and microfluidic studies inform compatibility but must not be presented as LSFM evidence when another imaging modality was used.

This review therefore does not attempt to cover every possible rhizosphere application of LSFM. Instead, it asks a narrower question: how can LSFM help researchers validate plant-microbe interaction mechanisms as live, spatially explicit processes (Downie et al., 2015; Etesami, 2026)? The answer requires more than imaging. LSFM can define the candidate interaction site, the order of events, and the geometry of contact, but functional interpretation must come from correlative and perturbation approaches such as biosensors, FISH, Raman-SIP, stable isotope probing, spatial omics, mutant comparisons, chemical perturbations, and targeted sampling (Huang et al., 2007; Cao et al., 2023; Schaible et al., 2022).

Existing reviews have established general LSFM design principles and performance limits (Power and Huisken, 2017; Stelzer et al., 2021), plant live-imaging practice (Berthet and Maizel, 2016; Ovecka et al., 2022), and microbial-community applications largely outside live root-microbe systems (Parthasarathy, 2018). Broader rhizosphere reviews synthesize multimodal sensing, imaging, modelling, microbial-identification, multi-omics, and spatial-omics strategies (Ahkami et al., 2024; Mbhele et al., 2026; Colombi et al., 2026). The present review does not duplicate these technology or methods surveys. Its distinct contribution is a claim-level evidence audit centred on live plant-microbe interactions: it separates direct LSFM observations from sample compatibility and correlative validation, translates four-dimensional images into root-centric arrival, residence, occupancy, growth, and persistence metrics, and specifies the evidence chain required to move from spatial co-occurrence to mechanism.

Literature published from 2004 through 2026 was identified through Web of Science Core Collection, CNKI Advanced Search, Google Scholar, and forward and backward citation tracing. Four predefined concept groups covered (i) LSFM and roots or the rhizosphere, (ii) LSFM and plant-microbe interactions, (iii) compatible sample and device platforms, and (iv) spatial validation methods; each was translated into platform-specific field syntax. The search was intended to support evaluation of LSFM-enabled plant-microbe claims, rather than pooled effect estimation or an exhaustive census of the literature. After the authors checked eligibility and full-text relevance, 51 records were retained. Exact queries, eligibility criteria, and source mappings are provided in **Supplementary File S1**.

Eligible records were peer-reviewed original articles or methods papers that used LSFM on live roots or plant-microbe systems, demonstrated a compatible sample or device, or supplied a spatially registered identity, activity, chemical, or functional validation method. Reviews were used only as contextual background. The quantitative classifications below therefore refer only to the 51 full-text records retained for this focused review. The author-proposed A-D framework is applied to one pre-specified representative claim extracted from each retained publication, rather than to an entire paper or imaging technology. The 51 retained publications therefore yielded 51 publication-representative-claim pairs. Level A applies when the biological observation was acquired directly with LSFM, SPIM, LST, or the LSFM-based plant-environment microscopy platform. Level B describes a compatible sample or device for which the biological observation was acquired with another modality. Level C covers identity, activity, chemistry, or function supplied by a correlative or endpoint method such as FISH, Raman-SIP, isotope tracing, a calibrated sensor, qPCR, omics, or flux measurement. Level D identifies a prospective plant-microbe application inferred from gaps in the 51-record set; it is a research opportunity and is not counted among the publication-representative-claim pairs.

Classification followed the measured variable and wording of each representative claim. The 51 publication-representative-claim pairs comprised three Level A, eight Level B, 37 Level C, and three contextual background units. These counts do not represent every statement made in each paper. The authors assigned the final levels from supporting full-text passages using the predefined criteria. Arbuscular mycorrhizal exchange, mature nodule nitrogen fixation, and multi-organism biocontrol competition are discussed separately as Level D research opportunities and do not enter this count. **Supplementary File S1** provides the decision rules, supporting passages, source routes, and complete 51-unit table.

## 2 LSFM platforms and experimental systems for rhizosphere research

LSFM is used here as the umbrella term for planar illumination with orthogonal or near-orthogonal fluorescence detection. Selective plane illumination microscopy (SPIM) is a specific LSFM configuration, light-sheet tomography (LST) denotes a root-oriented implementation, and plant-environment microscopy (PEM) is the particular large-volume environmental platform developed for transparent soil mesocosms; PEM is not a synonym for all LSFM systems. High-NA root SPIM or dual-view systems favour cellular and root-hair questions, whereas low-magnification, long-working-distance environmental systems trade single-cell resolution for millimetre-scale field of view. Open-top or oblique-plane geometries may reduce mounting constraints, but they have not yet been demonstrated in the reviewed live rhizosphere plant-microbe evidence set.

Instrument selection should therefore report illumination and detection geometry, numerical aperture, working distance, field of view, voxel sampling, volume time, multiview requirements, sample orientation, and spectral channels. A nominally thin sheet does not guarantee low phototoxicity: the relevant comparison depends on total delivered dose, illuminated volume, exposure frequency, reporter brightness, and experiment duration. Root autofluorescence, particle scattering, refractive-index mismatch, stripes and shadows, root-growth drift, photobleaching, channel cross-talk, fluorescent-protein maturation, and oxygen dependence can each bias the apparent spatial pattern. These limits require acquisition controls and must be balanced against biological realism rather than treated as generic advantages of LSFM.

LSFM illuminates a thin plane while collecting fluorescence along an approximately orthogonal detection axis. For a matched three-dimensional volume, this geometry can reduce out-of-focus excitation and accelerate volumetric sampling relative to point-scanning confocal microscopy (Huisken et al., 2004; Wu et al., 2013; Kumar et al., 2014), but the gain depends on sheet thickness, detection optics, view number, exposure, and reconstruction. In rhizosphere studies, the practical benefit is therefore not ‘low phototoxicity’ in the abstract; it is the ability to repeat sufficiently resolved volumes over the spatial and temporal scale needed to test a stated biological hypothesis.

The platform should be evaluated by the biological inference it permits. A useful LSFM experiment must preserve enough of the interaction environment to make microbial behaviour meaningful, provide optical access without eliminating the gradients that define the rhizosphere, and allow repeated imaging without detectable growth or microbial artefacts. Gel chambers, RootChip-style microdevices, transparent-soil analogues, and PEM satisfy different parts of this requirement (Grossmann et al., 2011; Downie et al., 2012; Massalha et al., 2017; Kaiser et al., 2023; Liu et al., 2021). Natural mineral soil cannot generally be imaged to equivalent depth by fluorescence LSFM; transparent polymers, refractive-index matching, temporary saturation, and thin chambers can alter hydraulics, oxygen, solute transport, and organism behaviour. Within the workflow in **Figure 1a**, optical access is therefore not merely a mounting requirement: it must keep labelled organisms spatially resolvable without stripping away the root-microbe-matrix context needed for interpretation.

**Figure 1:**
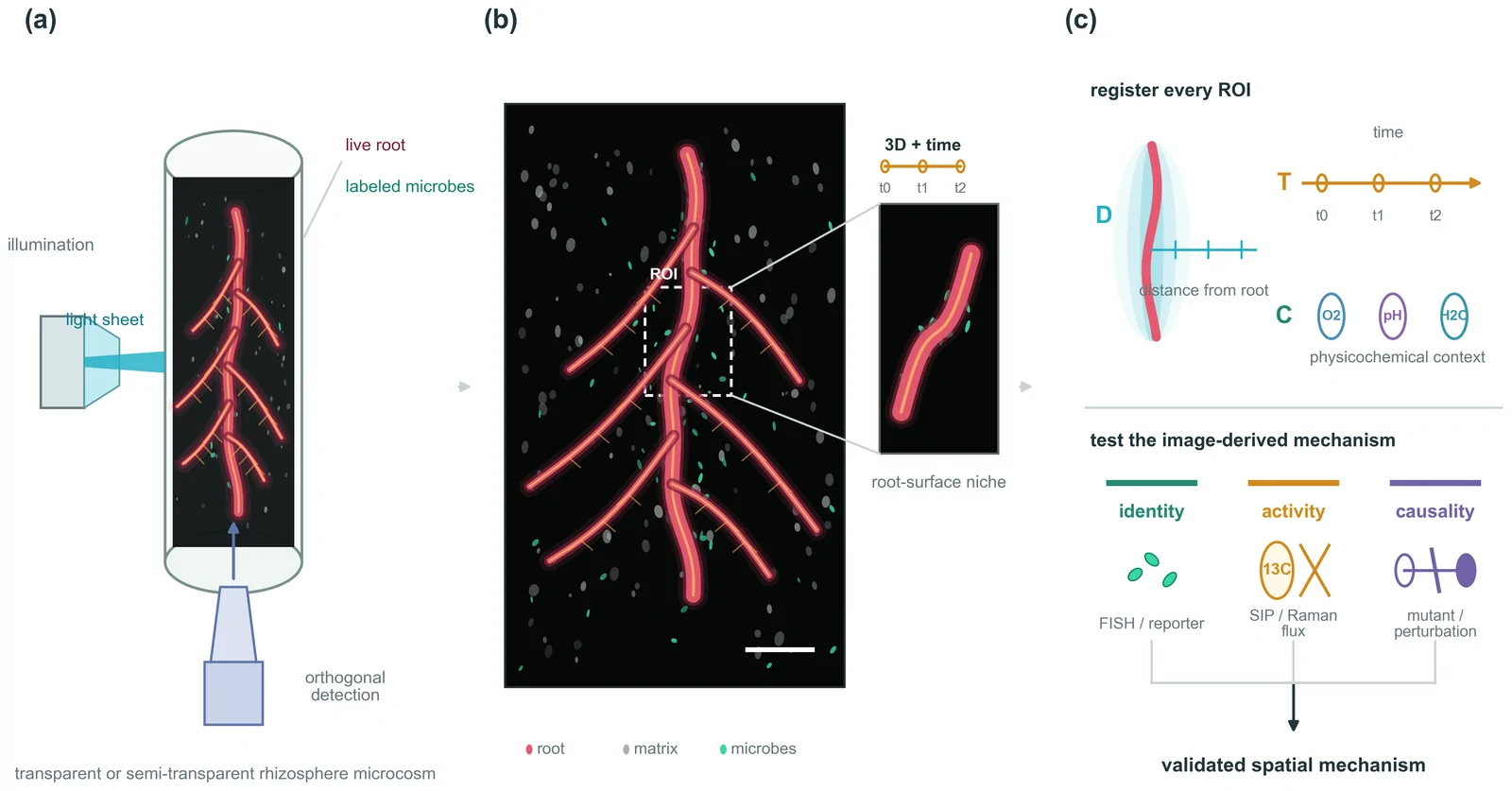
LSFM as a live spatial validation platform for rhizosphere plant-microbe interactions. **(a)** Optical access preserves a living root-microbe-matrix system. **(b)** Repeated multichannel volumes are registered to root-surface coordinates and image-derived ROIs. **(c)** Each ROI is indexed by distance, time, and physicochemical context and then linked to identity, activity, and perturbation assays. This is an author-generated conceptual model; not every connection is universal or supported by claim-matched Level A evidence in the verified full-text evidence set.

The main design choice is therefore not simply resolution or acquisition speed, but the evidence boundary created by the trade-off between biological realism and optical control (Downie et al., 2012; Grossmann et al., 2011; Kaiser et al., 2023). Agar or microfluidic systems provide high signal quality and precise inoculation but simplify soil structure, whereas transparent soils and plant-environment microscopy recover more pore-scale context while retaining important chemical and hydraulic differences from natural soils. **Table 1** compares these systems by their optical advantage, realism limitation, suitable application, and required validation. As illustrated in **Figure 1b**, once that compromise is chosen, repeated multichannel volumes must be registered to root-surface coordinates and converted into reproducible ROIs and trajectories; otherwise, improved optical access yields images but not comparable spatial evidence.

**Table 1:** Comparative experimental systems for live plant-microbe imaging and the validation requirements that bound LSFM inference.

Experimental system and representative evidence	Main optical advantage	Main realism limitation	Suitable application	Required validation
FEP/agarose chambers Meents et al. (2019); Hlaváčková et al. (2023), Level A	High spatial/temporal resolution with low optical background.	Simplified medium does not reproduce soil pore structure or chemistry.	Early infection, root-hair contact, and host signalling.	Genetic or chemical perturbation plus infection-stage, hormone, or host-response endpoint.
Microfluidic root devices Massalha et al. (2017), Level B	Defined flow and inoculation; repeated observation of one root zone.	Planar imposed-flow geometry simplifies gradients, pores, and dispersal.	Attachment, reversible colonisation, and pairwise competition.	Identity/viability testing, passive-flow control, and cue or strain perturbation.
Transparent-soil microcosms Sharma et al. (2020), Level B/C	Pore-like geometry and recoverable image-defined regions.	Index matching and synthetic particles alter hydraulics, oxygen, and chemistry.	Pore organisation and correlative isotope, chemical, or identity assays.	Fiducials, recovery yield/time lag, and an independent activity endpoint.
Plant-environment microscopy (PEM) Liu et al. (2021), Level A	Repeated millimetre-scale volumes connect roots, pores, and populations.	Population fluorescence does not resolve cell fate; transparent soil remains artificial.	First contact, root-distance trends, and pore-associated accumulation.	Cue/trait perturbation plus identity/viability and a matched functional measurement.
Natural soil and field linkage No claim-matched direct Level-A evidence	Highest ecological and translational relevance for functional outcomes.	Fluorescence LSFM lacks comparable penetration and repeat-volume access.	Testing persistence of an image-defined mechanism in realistic soil.	Independent functional endpoint, environmental replication, and field-reliability assessment.

Which experimental system can preserve the spatial event relevant to a plant-microbe question while remaining compatible with LSFM? This question should precede microscope selection because each preparation preserves a different part of the mechanism. A transparent microdevice can resolve attachment at a root hair, whereas a transparent-soil microcosm is better suited to movement through connected pores. A pathogen-biocontrol experiment additionally requires co-inoculation, repeated imaging, and later confirmation of organism identity or viability. Sample design is therefore part of the causal test rather than a technical preface (Grossmann et al., 2011; Downie et al., 2012; Liu et al., 2021; Kaiser et al., 2023).

The relevant evidence is the match between biological event and observable variable. Beneficial-microbe research provides the biological context for such tests (Finkel et al., 2017). Recruitment hypotheses predict where and when cells first approach the root in relation to exudate gradients, mucilage trails, root hairs, lateral-root emergence sites, wounds, and developmental zones. Rhizosphere syntheses show that recruitment chemistry varies with plant species, developmental stage, and stress (Shafi and Shahid, 2025; Baatsen et al., 2026; Liu, 2026). LSFM can test the resulting spatial prediction by measuring arrival trajectories, residence time, and the temporal order of microbial accumulation and plant response, although it cannot identify an unknown recruiting metabolite on its own. These measurements implement **Figure 1c**: each ROI is indexed by distance, time, and physicochemical context and then linked to organism identity, activity, and a perturbation test. The figure thus supports the central inferential boundary used throughout this review: LSFM can localise and order candidate interactions, whereas a mechanistic claim requires evidence beyond fluorescence proximity.

The same design principle applies across interaction types. Rhizobial invasion requires observation of host recognition, entry, infection-thread progression, cortical response, and nodule development, whereas AMF studies require access to hyphal approach, contact, penetration, and intracellular establishment (Jones et al., 2007; Oldroyd, 2013; Biswas et al., 2025; Shah et al., 2025). Pathogen and biocontrol studies instead need simultaneous access to the invader, antagonist, candidate entry site, and host response. These examples do not justify a single universal microcosm; they show that the preparation must expose the event that separates competing explanations.

No LSFM-compatible system simultaneously maximizes optical access, pore realism, environmental heterogeneity, and experimental control. The biological question should therefore determine the sample model and validation burden: a microdevice may establish local contact dynamics, whereas transparent soil or PEM can recover more pore context but still cannot establish field reliability. A convincing study should pre-specify the spatial prediction, include an informative perturbation, and validate the image-defined outcome with identity, viability, disease, or functional measurements (Nwokolo et al., 2021; Singh and Kumar, 2024; Khaskheli et al., 2025; Kanwar et al., 2026).

## 3 Microbial colonisation, biofilms, and root-surface niche formation

How does a microorganism progress from transient arrival to persistent root association, and which observations distinguish these states? Direct Level A evidence is currently represented mainly by population-scale bacterial dynamics in transparent-soil mesocosms. Evidence remains limited for single-cell fate, mature multispecies biofilms, and function in natural soil. Colonisation should therefore be treated as a set of measurable state changes, not as a synonym for fluorescence near a root. **Figure 2** organises these changes as a branching and reversible network linking arrival, attachment, microcolony formation, surface biofilm, dispersal, and endosphere entry rather than as a universal linear sequence.

**Figure 2:**
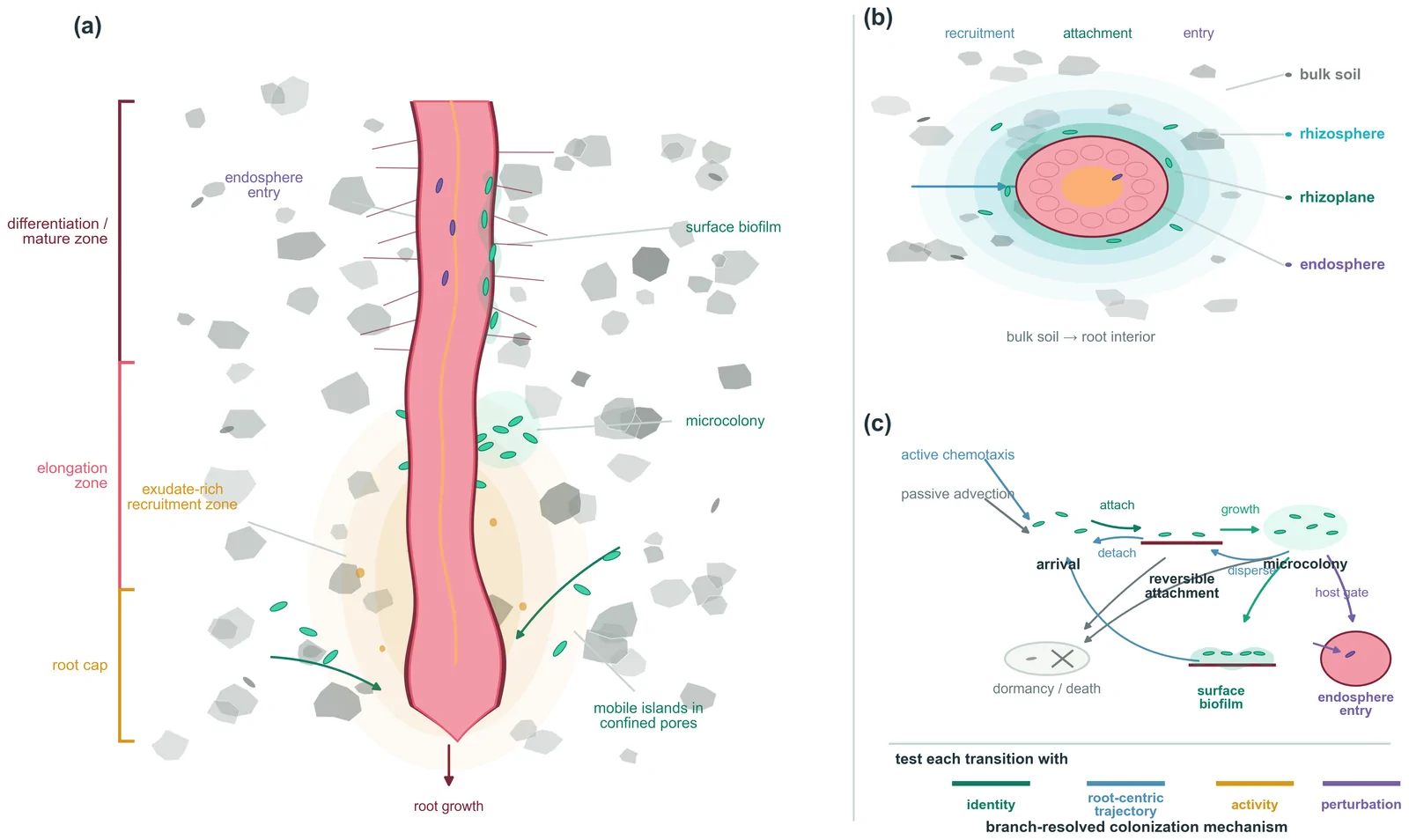
Branching and reversible states of bacterial recruitment and colonisation at the root-soil interface. **(a)** Longitudinal root zones and pore structure define candidate recruitment, attachment, microcolony, surface-biofilm, and entry sites. **(b)** Radial coordinates separate bulk soil, rhizosphere, rhizoplane, and endosphere. **(c)** Active chemotaxis and passive advection feed an arrival state; attachment can reverse, colonies can disperse, dormancy or death are explicit off-ramps, and surface biofilm and endosphere entry are distinct branches. Each transition requires identity, a root-centric trajectory, activity, and perturbation evidence. Some transitions in this conceptual framework lack claim-matched Level A support in the verified full-text evidence set.

The biological progression can be evaluated at three levels that are distinct from the claim-level A-D evidence framework. Presence means that labelled cells occur near or on a root. Spatial organisation requires reproducible enrichment at a root-centric site such as a root hair, lateral-root junction, pore, wound, or exudate-rich zone. Functional colonisation additionally requires activity and a host or community consequence. Movement among these states is not automatic: apparent approach can reflect active chemotaxis or passive transport, attachment may be followed by detachment, dormancy, death, growth, or dispersal, and a surface biofilm is not equivalent to endosphere entry. LSFM measures presence and organisation directly; residence, local growth, persistence, and an independent functional readout are needed to support establishment. A biofilm claim additionally requires time-resolved growth and persistence, matrix or reporter evidence, and an informative perturbation such as altered flow, matrix-gene mutation, or cue removal. Confocal studies of root microhabitats and large root sections provide complementary evidence for spatial organisation, but not direct LSFM evidence (Cardinale, 2014; Krzyzanowska et al., 2012).

In the first direct large-volume case, Liu et al. (2021) used a custom dual-illumination system to image approximately 3,600 mm³ of a lettuce seedling environment in Nafion transparent soil, with resolution as good as 5 µm and scans as frequent as every 30 min. GFP-labelled *B. subtilis* populations preferentially occupied small pores, approached the root cap as a first-contact zone, and accumulated around the root in pulses. The study linked bacterial density to root distance and pore size and checked that index-matching conditions did not abolish plant growth or bacterial motility. Within the **Figure 2** network, these observations support root-scale arrival and population accumulation, but the population-density signal did not resolve individual-cell trajectories, the chemical cue responsible for attraction, stable attachment, biofilm formation, or endosphere entry.

## 4 Symbiosis and pathogen interactions as dynamic spatial processes

Do the spatial rules derived from root colonisation also explain beneficial symbiosis and pathogen invasion? Both interaction types proceed through encounter, contact, entry or exclusion, and establishment, but they diverge in their host consequences. Treating them as boundary tests of a common spatial framework keeps the review focused: LSFM is used to determine where a trajectory begins, which route it follows, how rapidly stages occur, and whether a perturbation redirects that trajectory, rather than to catalogue every molecular pathway.

### 4.1 Direct evidence: rhizobial infection and fungal response

Hlaváčková et al. (2023) provide direct Level A evidence for an early rhizobial interaction. Intact alfalfa roots containing GFP-tagged stress-induced mitogen-activated protein kinase (SIMK) were imaged with mRFP-labelled *Ensifer meliloti* using dual-side LightSheet.Z1 acquisition at 0.466 × 0.466 × 1.497 µm sampling every 2 min for 80–120 min. LSFM resolved attachment, root-hair curling, infection-pocket formation, and relocation of GFP-SIMK toward infection sites. Genetically altered SIMK abundance, whole-mount immunofluorescence for activated MAPKs, and infection-thread counts supplied independent support. The evidence concerns early infection in an FEP/agarose chamber, not nitrogen fixation or performance in soil.

Meents et al. (2019) provide a second Level A case, but the directly imaged variable was the plant response rather than the fungal route. Arabidopsis roots carrying a dual auxin reporter were exposed to beneficial or pathogenic fungi and imaged on a LightSheet.Z1 every 30 min for 24 h. Beneficial *Piriformospora indica* increased reporter activity, whereas the pathogenic *Alternaria brassicicola* and *Verticillium dahliae* rapidly suppressed it. HPLC-MS phytohormone measurements and dose-response controls provided endpoint validation, while bleaching after 24 h defined an acquisition limit. The study supports time-resolved host signalling during root-fungus interaction; it does not directly establish fungal invasion geometry or long-term disease outcome.

### 4.2 Emerging applications: AMF and biocontrol

Within the 51-record verified full-text evidence set, no claim-matched Level A record supported arbuscular mycorrhizal exchange, mature nodule nitrogen fixation, or multi-organism antagonist-pathogen competition in soil-like matrices. These topics are therefore treated as Level D research opportunities, not as evidence units or statements of universal absence. Future experiments should pre-specify the observable stage, such as hyphal approach, penetration, infection-thread progression, antagonist arrival, or exclusion, and pair it with exchange, fixation, viability, disease, or host-defence endpoints. This narrower framing prevents the title and abstract from implying that every interaction class has already been demonstrated by LSFM.

This boundary is especially important for pathogen suppression and biocontrol. Plant-associated microbiomes can contribute to disease suppression through resource competition, induced host responses, and antimicrobial activity, but these roles remain context-dependent and require disease, viability, or host-defence endpoints in the same experimental framework (Zholdasbek et al., 2026). Accordingly, the Level D applications in this review are presented as testable routes rather than as established LSFM mechanisms.

Biocontrol and nematode-suppression models remain useful tests of the framework because they distinguish spatial interception from biological consequence (Kanwar et al., 2026). LSFM could determine whether an antagonist reaches an egg mass, invasion site, or pathogen entry route first, but fluorescence loss alone would not show killing or induced resistance. The minimum validation is a pre-specified spatial prediction, an antagonist or metabolite perturbation, and a matched endpoint such as pathogen viability, nematode activity, host-defence markers, or disease incidence.

## 5 From visualisation to functional validation

### 5.1 Operational definitions and statistical units

A registered root surface *S* provides the coordinate system for quantitative spatial validation. Radial distance is $d_{r}{(\text{x})=\text{min}}_{y\in S}\;\|x-y\|$, and root-surface geodesic distance is measured along the segmented surface rather than in a fixed laboratory frame. First-arrival time $T_{a}$ is the first frame in which signal exceeds a threshold derived from negative controls for a pre-specified number of consecutive frames. Dwell time is the summed occupied interval, colonised surface fraction is $A_{colonized}/A_{observed}$, aggregate growth is estimated as $\frac{d[ln(V)]}{dt}$ over a stated window, and persistence is reported as $\text{P}(T_{d}\;\geq \;\tau )$. A hotspot requires a defined spatial unit, time window, detection limit, and reproducible enrichment relative to a matched background; visual brightness alone is not an operational definition. These quantities form the measurement layer in **Figure 3a** and convert a visually defined hotspot into a testable spatial outcome.

**Figure 3:**
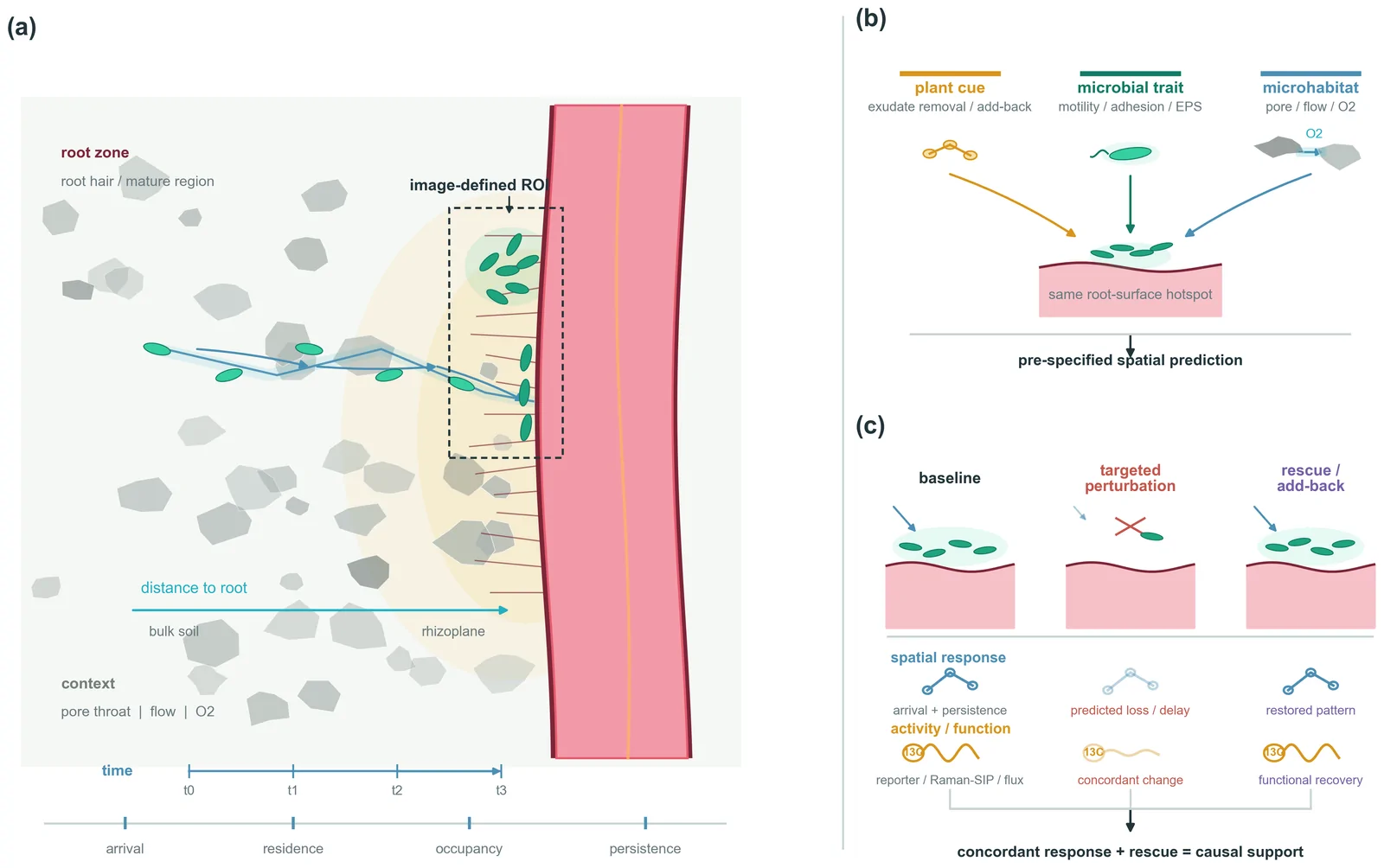
Causal validation framework for an image-defined root-surface hotspot. **(a)** LSFM quantifies arrival, dwell time, occupancy, growth, and persistence in a registered root-centric ROI. **(b)** Plant cues, microbial traits, or microhabitat conditions are perturbed against a pre-specified spatial prediction. **(c)** Baseline, perturbation, and rescue or add-back states are compared with an independent activity or functional endpoint. Concordant rescue strengthens causal support but is not, by itself, sufficient proof because off-target effects and alternative pathways remain possible. This is an author-generated conceptual model; not every link is supported by claim-matched Level A evidence in the verified full-text evidence set.

The experimental unit is an independently prepared plant-microcosm, and a biological replicate is an independent plant-microcosm established from a separate preparation. Multiple ROIs, root zones, time points, or cells within one microcosm are nested observations, not independent replicates. Analysis should therefore use hierarchical or mixed-effects models, or aggregate to the biological unit when justified; thresholds and tracking parameters should be fixed from controls or training data before treatment labels are revealed. Reports should include blinded scoring where feasible, root-growth registration, tracking error, missing-track rules, segmentation uncertainty, treatment allocation, and a pseudoreplication audit. Because the outcome is defined before treatment labels are examined, plant cues, microbial traits, or microhabitat conditions can then be perturbed against an explicit spatial prediction (**Figure 3b**), rather than selected retrospectively from the most conspicuous image.

### 5.2 Registration and correlative validation

Correlative validation occurs at three distinct levels. Simultaneous validation records a reporter, biosensor, or microsensor in the LSFM coordinate system. Sequential *in situ* validation returns the same specimen to a second modality using fiducials and a documented coordinate transform. Destructive endpoint validation fixes, sections, or physically recovers a registered region for CARD-FISH, FISH, Raman-SIP, NanoSIMS, qPCR, omics, chemistry, or flux analysis (Ferrari et al., 2006; Huang et al., 2007; Schaible et al., 2022). These levels should not be described as equally seamless because coordinate transfer, scale mismatch, sampling loss, and time delay increase from the first to the third.

A complete registration chain reports fiducial type and density, transformation model, landmark residuals or target registration error, ROI recovery yield, sample deformation, channel compatibility, fixation or sectioning steps, and elapsed time between imaging and validation. When a micrometre-scale hotspot cannot be recovered reliably, alternatives include a stable reporter, embedded microsensor, laser-capture strategy, or pre-registration of a larger sampling volume. The valid claim is then made at the recoverable scale rather than at the smallest visually apparent cluster. For causal interpretation, the same registered outcome is compared across baseline, perturbation, and rescue or add-back states and paired with an independent activity or functional endpoint (**Figure 3c**). Concordant rescue strengthens support but does not by itself exclude pleiotropy, off-target effects, or an unmeasured parallel pathway; orthogonal perturbations and alternative explanations remain necessary.

The following nitrogen-cycling scenario is a hypothetical worked example and a design template, not an existing LSFM demonstration. Rhizosphere priming and exudate-driven hotspots provide process context but do not themselves supply direct LSFM evidence (Cheng et al., 2014; Zhang et al., 2022). First, LSFM would identify a root-centric hotspot in which a labelled population coincides with a calibrated oxygen or nitrogen-responsive signal. Ratiometric sensor-foil imaging provides an independent precedent for spatial oxygen mapping in plant tissues and the rhizosphere (Tschiersch et al., 2012), but an LSFM reporter would still require its own calibration and interference controls. Second, a candidate cue or microbial trait would be perturbed while arrival, occupancy, and persistence are quantified in the same coordinate system. Third, a pre-registered sampling volume would be recovered for process-specific genes, Raman-FISH or Raman-SIP, stable-isotope tracing, microsensors, and N_2_O or other flux measurements (Huang et al., 2007; Sharma et al., 2020; Pang et al., 2021; Schaible et al., 2022). A nitrogen-transformation claim would be supported only if the spatial response and an independent process endpoint changed concordantly at the experimental-unit level; overlap among a root, cells, and a reporter would not be sufficient. **Table 2** operationalizes the **Figure 3** sequence by linking each claim to its observable, quantitative endpoint, minimum controls, independent validation, and explicit evidence limit.

**Table 2:** Operational evidence map for LSFM-enabled plant-microbe claims.

Claim	Direct LSFM observable	Quantitative endpoint	Minimum control	Independent validation	Evidence boundary
Recruitment	Approach trajectories and first accumulation in a root-centric zone	First-arrival time, radial/geodesic distance, directionality, and arrival rate	No-root or cue-deficient control; passive-flow estimate; fixed detection threshold	Cue chemistry, receptor or motility mutant, and identity assay	Approach does not distinguish chemotaxis from advection without perturbation
Residence and colonisation	Attachment, repeated occupancy, spread, and local growth	Dwell time, occupied fraction, colonised surface fraction, and growth rate	Viability control; matched root zone; independent plant-microcosms	Reporter expression, CFU or viability, competition, and host response	Repeated ROIs within one root are nested, not biological replicates
Biofilm	Persistent cluster architecture and expansion	Biovolume, growth, persistence probability, and dispersal	Matrix-deficient mutant or flow/cue perturbation; optical-overlap control	Matrix staining or reporter, resistance test, and viable-cell assay	Clustered fluorescence alone can reflect trapping or repeated arrival
Symbiotic or pathogenic response	Contact route, infection stage, and host reporter dynamics	Stage-transition time, penetration frequency, and reporter kinetics	Uninoculated host; compatible and incompatible partner; reporter calibration	Exchange, fixation, disease, viability, or host-defence endpoint	Contact or host signalling does not itself demonstrate exchange or disease
Correlative hotspot	Registered ROI selected from a live time series	ROI recovery yield, target registration error, and time lag	Fiducial-only and background ROI; blinded recovery	FISH, Raman-SIP, NanoSIMS, qPCR, omics, chemistry, or flux	The claim cannot be made at a finer scale than the recovered and registered sample
Causal perturbation	Spatial response to loss, addition, or rescue of a candidate driver	Pre-specified effect on arrival, occupancy, growth, or persistence	Vehicle, genotype background, off-target control, and independent replicate	Matched activity or functional endpoint and, where possible, orthogonal perturbation	Rescue strengthens causal support but is not sufficient proof on its own

Nitrogen fixation and phosphate mobilisation are agronomically important microbial functions, but they cannot be inferred from root-associated fluorescence or persistence alone. Future LSFM studies should therefore connect an image-defined hotspot to process-specific isotope, chemical, or flux measurements under realistic soil conditions (Rakhmatova et al., 2026).

## 6 Challenges and future priorities

Current LSFM applications should be interpreted within four linked limits. First, direct claim-matched Level A evidence is restricted to three examples, so it cannot yet support broad statements about rhizosphere function. Second, the optical access that permits repeated volumes commonly depends on agarose, microdevices, or transparent soils that simplify natural pore structure, chemistry, water, and oxygen conditions. Third, direct multispecies and mature-biofilm studies remain scarce. Finally, microbial presence or persistence under controlled conditions does not demonstrate a stable functional effect after transfer to heterogeneous soil or field settings, where formulation, delivery, host context, and environmental variation can alter performance (Atav, 2026). These limits do not diminish the value of LSFM; they specify the independent validation required for a defensible claim.

Progress in rhizosphere LSFM will depend on experimental systems that can be compared across studies and on reporters that distinguish presence from activity. Reports should specify medium composition, optical properties, inoculation method, root age, imaging interval, light dose, microbial strain, and post-imaging validation. Distance, time, and context from the SERS framework offer a practical structure for this information (Etesami, 2026), consistent with the broader need for standardised efficacy protocols in field-oriented plant growth-promoting bacteria (PGPB) research (Valenzuela et al., 2025). Reporter panels can combine constitutive strain labels with promoter, calcium, hormone, viability, exopolysaccharide, or physicochemical sensors, provided that phototoxicity, growth effects, spectral overlap, and altered strain behaviour are tested.

An equally important requirement is a reproducible link between the live image and the assay used to interpret it. Simultaneous, sequential *in situ*, and destructive endpoint workflows should be distinguished and accompanied by fiducials, registration error, recovery yield, and time lag. Transparent-soil systems can support this transfer, whereas confocal or Raman results remain Level B or C unless LSFM acquisition is demonstrated (Sharma et al., 2020; Schaible et al., 2022). Open analysis is essential because conclusions depend on registration, segmentation, tracking, detection thresholds, and hierarchical sampling. **Table 3** summarizes the minimum reporting requirements, and **Figure 4** maps the required sequence: formulate a spatial prediction in a system with sufficient optical access, test it with an independent functional assay, and then assess whether it holds in more realistic soil or field contexts.

**Table 3:** Minimum reporting checklist for rhizosphere plant-microbe LSFM studies.

Domain	Minimum items	Reporting expectation
Biological material	Plant species, genotype, age, root zone; microbial strain, inoculum, viability, and label	State the biological experimental unit and the number of independent plant-microcosms
Matrix and environment	Medium, substrate, particle or pore distribution, water content, refractive-index matching, oxygen, pH, temperature, and flow	Report how optical preparation may alter hydraulics, chemistry, oxygen, or organism behaviour
Optical system	LSFM configuration, illumination/detection geometry, objectives, NA, working distance, sheet thickness, field of view, and channels	Identify commercial or custom components and distinguish LSFM from adjacent modalities
Acquisition and dose	Voxel size, volume dimensions, exposure, laser power at sample, interval, duration, views, and total light dose	Provide controls for growth, motility, bleaching, phototoxicity, and fluorescent-protein maturation
Spectral and calibration controls	Single-colour controls, autofluorescence, cross-talk, flat field, point-spread function, intensity standard, and detection limit	Pre-specify channel unmixing and the threshold source
Registration	Root-centric coordinate system, fiducials, transformation model, residual error, drift correction, and ROI recovery yield	Report the scale and time lag at which live and endpoint measurements can legitimately be linked
Image analysis	Software/version, preprocessing, segmentation, tracking, manual edits, missing-track rule, and uncertainty	Share code, parameter files, representative raw data, masks, and exclusions
Operational definitions	Arrival, dwell time, occupancy, colonised fraction, aggregate growth, hotspot, and persistence	Give equations, units, spatial scale, time window, and consecutive-frame rule
Design and statistics	Randomization, blinding, treatment allocation, biological n, nested ROIs, model, effect size, and multiple testing	Treat the plant-microcosm as the experimental unit and audit pseudoreplication
Functional validation	Identity, activity, perturbation, rescue/add-back, and process endpoint	State evidence level A-D and keep the claim within the weakest required validation step

**Figure 4:**
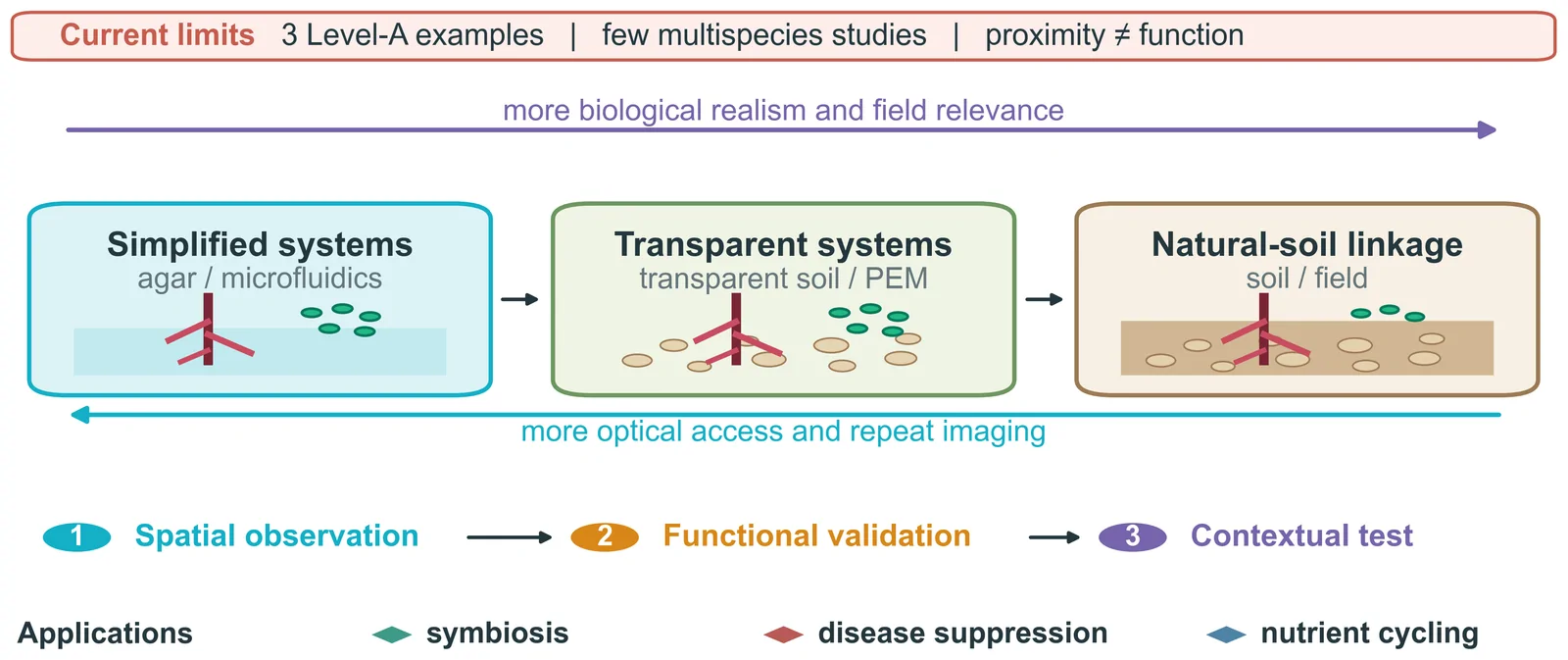
Evidence pathway from live imaging to field-relevant inference.

**Figure 4** places this sequence within the experimental-system trade-off. Moving from simplified live systems (agar/gel or microfluidic devices) through transparent systems to natural-soil or field linkage increases biological realism and field relevance but reduces optical access and repeat LSFM volume. LSFM can therefore resolve root-centric arrival, occupancy, and persistence in compatible systems, whereas an image-derived spatial pattern remains a hypothesis until it is tested with matched identity/activity and perturbation evidence, and then with soil- or plant-function outcomes and context replication. This staged logic is relevant to symbiosis, disease suppression, and nutrient cycling.

## 7 Conclusion

Direct LSFM evidence for live plant-microbe interactions remains limited to three claim-level examples. It already shows where the method is informative: locating recruitment onset and resolving early infection-associated or population-level changes after initial contact. It does not yet establish arbuscular mycorrhizal exchange, mature nodule function, multispecies biocontrol, or field reliability. In these settings, LSFM should be used to test a pre-specified spatial prediction in a mechanism-matched model, followed by registered identity, activity, perturbation, and functional measurements rather than by inference from fluorescence proximity alone.

Study design should begin with a spatial prediction, then select the least simplifying system that can resolve it. Root-centric measures of arrival, residence, growth, persistence, and branching outcomes provide a common analytical basis, provided that nested ROIs are not treated as independent biological replicates. Linking live ROIs to simultaneous, sequential *in situ*, or destructive endpoint assays—and reporting their registration error—sets the boundary between spatial observation, functional support, and field-relevant inference.

## Glossary

LSFM: light-sheet fluorescence microscopy; an umbrella term for planar illumination with orthogonal or near-orthogonal fluorescence detection
SPIM: selective plane illumination microscopy; a specific LSFM configuration rather than a separate evidence category
PEM: plant-environment microscopy; a custom large-volume LSFM platform for imaging plants, microorganisms, and transparent-soil structure
ROI: region of interest defined in a registered root-centric coordinate system
FISH: fluorescence *in situ* hybridization for spatially resolved taxonomic or gene-target identity, usually after fixation
SIP/Raman-SIP: stable-isotope probing, with Raman-SIP detecting isotope incorporation in recovered cells; it is distinct from the SERS sampling framework
SERS: Spatially Explicit, Resolved, and Situationally contextualized sampling; here the acronym does not mean surface-enhanced Raman spectroscopy
DTC: distance, time, and context; the minimum spatial-temporal metadata used to interpret a rhizosphere interaction
Live spatial validation: live imaging defines where and when a candidate interaction occurs, and independent assays test identity, activity, causality, or function
Interaction hotspot: a predefined spatial and temporal unit with reproducible enrichment above a controlled threshold; visual brightness alone is insufficient
Correlative validation: simultaneous, sequential *in situ*, or destructive endpoint assays linked to an image-defined ROI with reported registration error
