# Supplementary material for: Live spatial validation of plant-microbe interactions in the rhizosphere with light-sheet fluorescence microscopy
Source: Front Plant Sci. 2026 Oct 5;17:1953583. doi: 10.3389/fpls.2026.1953583 (PMC13679359; doi:10.3389/fpls.2026.1953583)
Supplement: Supplementary file 1 [file DataSheet1.docx]

**Supplementary File S1. Search documentation and claim-level evidence audit**

*Live spatial validation of plant-microbe interactions in the rhizosphere with light-sheet fluorescence microscopy*

# S1. Scope and source documentation

Literature published from 2004 through 2026 was identified for the evidence corpus. Web of Science Core Collection and CNKI Advanced Search were the main database sources; Google Scholar and forward/backward citation tracing provided supplementary discovery and title/DOI verification. The same four predefined concepts were translated into each platform's field and operator syntax. Query strings, interface settings, eligibility criteria, and source routes are documented for transparency. This documentation supports transparent construction of the author-verified evidence corpus used in this focused Review.

# S2. Search concepts and corpus construction

Literature was identified across Web of Science Core Collection, CNKI Advanced Search, Google Scholar, and forward/backward citation routes. After author verification of eligibility and full-text relevance, 51 records formed the evidence corpus. These records yielded 51 paper-derived claim units (A = 3, B = 8, C = 37, Context = 3). Prospective Level D applications are discussed separately and are not counted as verified evidence units.

Table S1. Search concepts, operational API strings, settings, and role in the review.

| **Source / group** | **Verbatim query** | **Settings** | **Role in this review** |
| --- | --- | --- | --- |
| Web of Science Core Collection Q1 | TS=(("light sheet" OR "light-sheet fluorescence microscopy" OR "selective plane illumination microscopy" OR SPIM OR "light-sheet tomography") AND (root* OR rhizosphere)) | Field: Topic; span: 2004-2026; sort: Relevance; language: No language restriction; expansion: Database default | Database discovery route; author-verified records contributed to the evidence corpus |
| Web of Science Core Collection Q2 | TS=(("light sheet" OR "light-sheet fluorescence microscopy" OR "selective plane illumination microscopy" OR SPIM OR "light-sheet tomography" OR "plant-environment microscopy") AND (bacter* OR fung* OR rhizobi* OR symbio* OR pathogen* OR "plant-microbe")) | Field: Topic; span: 2004-2026; sort: Relevance; language: No language restriction; expansion: Database default | Database discovery route; author-verified records contributed to the evidence corpus |
| Web of Science Core Collection Q3 | TS=(("plant-environment microscopy" OR "transparent soil" OR RootChip OR microfluidic*) AND (root* OR rhizosphere) AND (imaging OR microscopy)) | Field: Topic; span: 2004-2026; sort: Relevance; language: No language restriction; expansion: Database default | Database discovery route; author-verified records contributed to the evidence corpus |
| Web of Science Core Collection Q4 | TS=((root* OR rhizosphere) AND ("oxygen sensor" OR optode OR microsensor OR "stable isotope" OR "Raman-SIP" OR FISH OR NanoSIMS) AND (spatial OR imaging OR registration)) | Field: Topic; span: 2004-2026; sort: Relevance; language: No language restriction; expansion: Database default | Database discovery route; author-verified records contributed to the evidence corpus |
| Google Scholar Q1 | ("light sheet" OR "light-sheet fluorescence microscopy" OR "selective plane illumination microscopy" OR SPIM OR "light-sheet tomography") (root OR roots OR rhizosphere) | Field: Platform default; span: 2004-2026; sort: Relevance; language: No language restriction; expansion: Not configurable | Supplementary discovery and title/DOI-verification route; author-verified records contributed to the evidence corpus |
| Google Scholar Q2 | ("light sheet" OR "light-sheet fluorescence microscopy" OR "selective plane illumination microscopy" OR SPIM OR "light-sheet tomography" OR "plant-environment microscopy") (bacteria OR bacterial OR fungus OR fungi OR rhizobium OR symbiosis OR pathogen OR "plant-microbe") | Field: Platform default; span: 2004-2026; sort: Relevance; language: No language restriction; expansion: Not configurable | Supplementary discovery and title/DOI-verification route; author-verified records contributed to the evidence corpus |
| Google Scholar Q3 | ("plant-environment microscopy" OR "transparent soil" OR RootChip OR microfluidic) (root OR roots OR rhizosphere) (imaging OR microscopy) | Field: Platform default; span: 2004-2026; sort: Relevance; language: No language restriction; expansion: Not configurable | Supplementary discovery and title/DOI-verification route; author-verified records contributed to the evidence corpus |
| Google Scholar Q4 | (root OR roots OR rhizosphere) ("oxygen sensor" OR optode OR microsensor OR "stable isotope" OR "Raman-SIP" OR FISH OR NanoSIMS) (spatial OR imaging OR registration) | Field: Platform default; span: 2004-2026; sort: Relevance; language: No language restriction; expansion: Not configurable | Supplementary discovery and title/DOI-verification route; author-verified records contributed to the evidence corpus |
| CNKI Q1 | 主题=(光片显微镜+光片荧光显微镜+选择性平面照明显微镜+LSFM+SPIM+光片层析) AND 主题=(根+根系+根际) | Field: Subject; span: All years; sort: Relevance; language: Chinese and English records; expansion: Chinese-English expansion disabled | Database discovery route; author-verified records contributed to the evidence corpus |
| CNKI Q2 | 主题=(光片显微镜+光片荧光显微镜+选择性平面照明显微镜+LSFM+SPIM+植物环境显微镜) AND 主题=(细菌+真菌+根瘤菌+共生+病原+植物微生物互作+植物-微生物相互作用) | Field: Subject; span: All years; sort: Relevance; language: Chinese and English records; expansion: Chinese-English expansion disabled | Database discovery route; author-verified records contributed to the evidence corpus |
| CNKI Q3 | 主题=(植物环境显微镜+透明土壤+根芯片+RootChip+微流控+微流体) AND 主题=(根+根系+根际) AND 主题=(成像+显微) | Field: Subject; span: All years; sort: Relevance; language: Chinese and English records; expansion: Chinese-English expansion disabled | Database discovery route; author-verified records contributed to the evidence corpus |
| CNKI Q4 | Q4a 主题=(根+根系+根际)*(氧传感器+氧敏探针+平面光极+optode+微传感器); Q4b 主题=(根+根系+根际)*(稳定同位素成像+同位素成像+拉曼稳定同位素探针+"Raman-SIP"); Q4c 主题=(根+根系+根际)*(荧光原位杂交+"fluorescence in situ hybridization"+NanoSIMS+纳米二次离子质谱) | Field: Subject; recall-oriented method-family sensitivity split; span: All years; sort: Relevance; language: Chinese and English records; expansion: Chinese-English expansion disabled | Database discovery route; author-verified records contributed to the evidence corpus |

# S3. Construction of the verified evidence corpus

Figure S1. Construction of the author-verified evidence corpus. Database and citation routes supported literature discovery; AI-assisted recommendations were limited to preliminary organization, and the authors made all eligibility, inclusion, interpretation, and classification decisions. The final 51-record corpus supported the claim-level evidence audit. Prospective applications are discussed separately and are not counted as evidence units.

# S4. Eligibility and author verification

Peer-reviewed original articles and methods papers were eligible when they directly used LSFM in a live root or plant-microbe system, demonstrated an immediately compatible sample/device role, or supplied a spatially registered identity, activity, chemical, or functional validation method. Reviews were used only as Context. AI-assisted recommendations supported preliminary literature organization; every eligibility decision, source interpretation, and final inclusion was checked and confirmed by the authors. No automated recommendation was treated as a final evidence judgement. All quantitative classifications are limited to the 51 author-verified full-text records.

# S5. A-D claim-level classification rules

The unit of classification is the claim, not the paper. Boundary examples: Meents et al. (2019) are A for imaged plant reporter dynamics but not for the unobserved fungal route; Sharma et al. (2020) are B for sample compatibility and C only for a separately stated Raman-SIP endpoint; Hlaváčková et al. (2023) are A for early rhizobial events but not for mature nodule nitrogen fixation. Claims spanning levels were narrowed or assigned to the weaker level. The authors assigned evidence levels from full-text supporting passages using the predefined rules in Table S2. The final 51 units comprised A = 3, B = 8, C = 37, and Context = 3. Level D is reserved for prospective research opportunities derived from verified evidence gaps and is not included in the evidence total.

Table S2. Mutually exclusive A-D claim-level decision rules.

| **Level** | **Mutually exclusive decision rule for one claim** |
| --- | --- |
| A | The biological observation asserted in the claim was acquired directly with LSFM, SPIM, LST, or the LSFM-based plant-environment microscopy platform. |
| B | The claim concerns an LSFM-compatible sample or device, but the biological observation was demonstrated using another modality. |
| C | The claim concerns identity, activity, chemistry, or function supplied by an independent correlative, sensor, isotope, molecular, omics, or flux method. |
| D | A prospective plant-microbe application derived from gaps in the author-verified evidence corpus; it is not counted as a verified evidence unit. |
| Context | A review or methodological guidance record used for framing but not counted as direct, enabling, or correlative evidence. |

# S6. Positioning against the closest reviews

The closest reviews are complementary rather than interchangeable with the present article. They address general LSFM operation, plant live imaging, microbial communities outside the rhizosphere, broad rhizosphere technology portfolios, identification methods, or soil imaging-spatial omics. None applies a claim-level A-D audit to live plant-microbe LSFM evidence while also defining root-centric temporal metrics and a spatial causality chain. Table S3 makes this distinction explicit.

Table S3. Scope comparison with the closest reviews.

| **Review** | **Primary scope** | **Distinct role of the present review** |
| --- | --- | --- |
| Berthet and Maizel (2016) | Plant LSFM preparation and live-imaging practice | Audits plant-microbe claims and links images to causal validation. |
| Power and Huisken (2017) | General multiscale LSFM design and implementation | Focuses on rhizosphere evidence boundaries and root-centric metrics. |
| Parthasarathy (2018) | LSFM monitoring of microbial communities in biofilms, hosts, and model systems | Restricts inference to live root-microbe interactions and registered validation. |
| Stelzer et al. (2021) | General LSFM principles, performance, and applications | Uses a claim-level A-D evidence audit rather than a technology primer. |
| Ahkami et al. (2024) | Multiscale rhizosphere sensing, imaging, and computation | Defines what LSFM directly demonstrates versus what other modalities validate. |
| Mbhele et al. (2026) | Systematic comparison of rhizosphere bacterial and fungal identification methods | Centres live spatial dynamics and causal perturbation, not identification alone. |
| Colombi et al. (2026) | Integration of soil imaging and spatial omics for root-soil interactions | Adds time-resolved plant-microbe evidence levels and arrival-to-persistence metrics. |

# S7. Author-verified evidence corpus and source traceability

Table S4. Author-verified claim-level evidence corpus, evidence level, and source route.

| **Claim / citation** | **Level** | **DOI** | **Retrieval source / query** | **Claim-specific role** |
| --- | --- | --- | --- | --- |
| CLM-0001 - Strömberg (2008) | C | 10.1021/es071400q | Web of Science Core Collection; Q4 | Planar-optode mapping of rhizosphere chemistry; a potential spatial endpoint for registered LSFM ROIs. |
| CLM-0002 - Ma et al. (2019) | C | 10.1016/j.soilbio.2019.06.001 | Web of Science Core Collection; Q4 | Spatial zymography paired with pH mapping; useful for checking enzyme activity and chemistry at image-defined hotspots. |
| CLM-0003 - Rodionov et al. (2020) | C | 10.1007/s10533-020-00700-8 | Web of Science Core Collection; Q4 | NanoSIMS nutrient mapping that could link registered rhizosphere locations to elemental or isotopic endpoints. |
| CLM-0004 - Rudolph-Mohr et al. (2021) | C | 10.1007/s11104-021-05120-7 | Web of Science Core Collection; Q4 | Correlative measurement of root architecture and rhizosphere chemistry at matched spatial scales. |
| CLM-0005 - Engelhardt et al. (2024) | B | 10.1099/mic.0.001477 | Web of Science Core Collection; Q3 | Confined-space rhizosphere colonisation imaged without LSFM; evidence that the platform geometry can support live observation. |
| CLM-0006 - Noirot-Gros et al. (2020) | B | 10.3389/fpls.2020.00408 | Web of Science Core Collection; Q3 | Microfluidic live imaging of tree root-microbe interactions; device feasibility shown with a non-LSFM modality. |
| CLM-0007 - Ge et al. (2021) | Context | 10.1007/s11104-021-05133-2 | Web of Science Core Collection; Q3 | Methods context for laser manipulation in transparent soil; excluded from direct plant-microbe evidence. |
| CLM-0008 - Scholz et al. (2019) | C | 10.1093/femsec/fiz062 | Web of Science Core Collection; Q4 | FISH combined with electrical microsensors; an identity and physicochemical check for selected rhizosphere locations. |
| CLM-0009 - Rodeghiero et al. (2018) | C | 10.3389/fmicb.2018.01491 | Web of Science Core Collection; Q4 | Optode measurements of oxygen dynamics during root-pathogen interaction; a spatial chemistry endpoint independent of LSFM. |
| CLM-0010 - Hlavácková et al. (2023) | A | 10.1093/jxb/erad111 | Web of Science Core Collection; Q1; Q2 | Direct LSFM evidence for rhizobial attachment and early infection-associated events in root hairs. |
| CLM-0011 - Bandara et al. (2021) | C | 10.3389/fpls.2021.668929 | Web of Science Core Collection; Q4 | A correlative workflow combining rhizosphere identity with local chemistry at matched sampling locations. |
| CLM-0012 - Koop-Jakobsen et al. (2015) | C | 10.1007/s12237-014-9861-y | Web of Science Core Collection; Q4 | Planar-optode oxygen maps that could validate chemical conditions around registered root regions. |
| CLM-0013 - Walton et al. (2022) | C | 10.1021/jasms.2c00081 | Web of Science Core Collection; Q3 | Spatial metabolite imaging; an independent molecular map for image-selected rhizosphere regions. |
| CLM-0014 - Vidal et al. (2018) | C | 10.3389/fenvs.2018.00009 | Web of Science Core Collection; Q4 | Three-dimensional soil imaging coupled to NanoSIMS, linking structure with carbon-transfer measurements. |
| CLM-0015 - Gamez et al. (2020) | C | 10.1007/s00248-020-01571-0 | Web of Science Core Collection; Q4 | FISH and confocal mapping of root colonisation; suitable for confirming taxon identity and spatial occupancy. |
| CLM-0016 - Lenzewski et al. (2018) | C | 10.1111/nph.14973 | Web of Science Core Collection; Q4 | Dual-gas planar-optode measurements for resolving coupled chemical gradients around roots. |
| CLM-0017 - Bellgard et al. (2016) | C | 10.1111/efp.12280 | Web of Science Core Collection; Q4 | Pathogen-specific FISH for confirming organism identity at image-defined infection sites. |
| CLM-0018 - Parashar et al. (2011) | B | 10.1063/1.3604788 | Web of Science Core Collection; Q3 | A microfluidic plant-pathogen platform demonstrated with another imaging modality; relevant to future LSFM adaptation. |
| CLM-0019 - Ge et al. (2025) | C | 10.3390/toxics14010034 | Web of Science Core Collection; Q4 | Planar-optode mapping of rhizosphere microecology, providing an independent spatial chemistry readout. |
| CLM-0020 - Gorka et al. (2019) | C | 10.3389/fmicb.2019.00168 | Web of Science Core Collection; Q4 | Stable-isotope tracing of carbon transfer; a functional endpoint for spatially selected regions. |
| CLM-0021 - Fettrow et al. (2026) | C | 10.1016/j.soilbio.2026.110149 | Web of Science Core Collection; Q4 | Integrated optode, microbial, and gas-flux measurements for connecting local chemistry with process rates. |
| CLM-0022 - O'Callaghan et al. (2018) | B | 10.1038/s41598-017-18797-7 | Web of Science Core Collection; Q1; Q3 | Live imaging of rhizosphere biota in transparent soil; sample compatibility established without LSFM acquisition. |
| CLM-0023 - Liu et al. (2025) | Context | 10.1016/j.biosystemseng.2025.02.006 | Web of Science Core Collection; Q1; Q2 | Methods context for designing an LSFM-compatible plant-soil microcosm; not counted as direct biological evidence. |
| CLM-0024 - Fay et al. (2024) | Context | 10.1007/s11368-023-03650-4 | Web of Science Core Collection; Q4 | Methods context for protecting planar pH optodes during long-term soil monitoring and releasing them for optical readout. |
| CLM-0025 - Lohse et al. (2021) | C | 10.3389/fpls.2021.753812 | Web of Science Core Collection; Q4 | Mass-spectrometry imaging of rhizosphere metabolites; an endpoint map for selected root-associated regions. |
| CLM-0026 - Koop-Jakobsen et al. (2018) | C | 10.3389/fpls.2018.00541 | Web of Science Core Collection; Q4 | Multi-analyte optode mapping for comparing chemical gradients across registered rhizosphere locations. |
| CLM-0027 - Larsen et al. (2015) | C | 10.1007/s11104-015-2382-z | Web of Science Core Collection; Q4 | Planar-optode measurements of oxygen dynamics around roots; an independent chemical validation route. |
| CLM-0028 - Holz et al. (2020) | C | 10.1016/j.rhisph.2020.100266 | Web of Science Core Collection; Q4 | Planar-optode CO2 mapping for testing local respiratory patterns at image-defined regions. |
| CLM-0029 - Massalha et al. (2017) | B | 10.1073/pnas.1618584114 | Web of Science Core Collection; Q3 | TRIS microfluidic imaging of live root-bacteria dynamics; platform feasibility demonstrated by confocal microscopy rather than LSFM. |
| CLM-0030 - Patko et al. (2024) | C | 10.1007/s11104-023-06151-y | Web of Science Core Collection; Q1 | Smart-soil measurements of pH gradients, offering a chemical reference for registered root zones. |
| CLM-0031 - Cao et al. (2023) | C | 10.1038/s41467-023-37188-3 | Web of Science Core Collection; Q4 | Sequential FISH profiling for assigning microbial identity and spatial organisation to selected regions. |
| CLM-0032 - Clode et al. (2009) | C | 10.1104/pp.109.141499 | Web of Science Core Collection; Q4 | NanoSIMS mapping of nutrient uptake; a cell-scale elemental endpoint for correlative analysis. |
| CLM-0033 - Schmidt et al. (2023) | C | 10.1111/nph.19112 | Web of Science Core Collection; Q4 | Gold-FISH targeted NanoSIMS, combining taxonomic identification with isotope or elemental measurements. |
| CLM-0034 - Mayerhofer et al. (2021) | C | 10.1111/nph.17591 | Web of Science Core Collection; Q4 | NanoSIMS isotope imaging of ectomycorrhiza, linking spatial structure to nutrient transfer. |
| CLM-0035 - Lu et al. (2007) | C | 10.1111/j.1462-2920.2006.01164.x | Web of Science Core Collection; Q4 | Spatial stable-isotope probing for identifying active populations in defined sample regions. |
| CLM-0036 - Jones et al. (2013) | C | 10.1111/nph.12405 | Web of Science Core Collection; Q4 | NanoSIMS analysis of plant-bacteria nitrogen competition at cellular spatial resolution. |
| CLM-0037 - Aufrecht et al. (2018) | B | 10.1002/adbi.201800048 | Web of Science Core Collection; Q3 | Microfluidic imaging of root-colonisation dynamics with a non-LSFM modality; a device-level feasibility example. |
| CLM-0038 - Downie et al. (2012) | B | 10.1371/journal.pone.0044276 | Web of Science Core Collection; Q3 | Transparent-soil imaging of root-bacteria interactions; matrix compatibility demonstrated without LSFM. |
| CLM-0039 - Kreuzeder et al. (2018) | C | 10.1007/s11104-017-3542-0 | Web of Science Core Collection; Q4 | Sub-millimetre mapping of phosphorus biogeochemistry for validating chemistry around selected root regions. |
| CLM-0040 - Li et al. (2024) | C | 10.1016/j.jenvman.2023.119763 | Web of Science Core Collection; Q4 | Combined phosphorus and oxygen imaging for linking registered rhizosphere structure to local chemistry. |
| CLM-0041 - Honeker et al. (2016) | C | 10.1016/j.mimet.2016.09.018 | Web of Science Core Collection; Q4 | Correlative FISH and micro-XRF for connecting microbial identity with elemental distributions. |
| CLM-0042 - Cao et al. (2026) | C | 10.1016/j.crmeth.2026.101381 | Web of Science Core Collection; Q4 | Automated sequential FISH for spatially resolved identification of root-associated microbiome members. |
| CLM-0043 - Fang et al. (2021) | C | 10.1021/acs.est.1c05358 | Web of Science Core Collection; Q4 | Correlative phosphorus and oxygen imaging, providing paired chemical endpoints for selected rhizosphere regions. |
| CLM-0044 - Spohn et al. (2015) | C | 10.1016/j.soilbio.2015.06.018 | Web of Science Core Collection; Q4 | Zymography paired with bacterial spatial mapping to relate enzyme activity to microbial distribution. |
| CLM-0045 - Watt et al. (2006) | C | 10.1111/j.1462-2920.2005.00973.x | Web of Science Core Collection; Q4 | FISH-based quantification of native root bacteria for identity and abundance checks at sampled locations. |
| CLM-0046 - Liu et al. (2021) | A | 10.1073/pnas.2109176118 | Web of Science Core Collection; Q1; Q2; Q3 | Direct plant-environment LSFM evidence for population-scale Bacillus dynamics across a transparent-soil rhizosphere. |
| CLM-0047 - Mittmann-Goetsch et al. (2024) | C | 10.1007/s13157-024-01867-8 | Web of Science Core Collection; Q4 | Spatial redox and pH measurements for testing root-driven chemical gradients. |
| CLM-0048 - Meents et al. (2019) | A | 10.3389/fmicb.2019.00380 | Web of Science Core Collection; Q1; Q2 | Direct LSFM measurement of root auxin-reporter responses during fungal interaction; fungal invasion itself was not imaged. |
| CLM-0049 - Chen et al. (2018) | C | 10.1264/jsme2.me18010 | Web of Science Core Collection; Q4 | Stable-isotope and micro-XRF analysis of mycorrhizal systems, linking nutrient transfer with elemental distributions. |
| CLM-0050 - Quinn et al. (2025) | B | 10.1093/pnasnexus/pgaf118 | Web of Science Core Collection; Q4 | A transparent mineral scaffold for rhizosphere microbes; material compatibility shown with a non-LSFM imaging approach. |
| CLM-0051 - Blossfeld et al. (2013) | C | 10.1093/aob/mct047 | Web of Science Core Collection; Q4 | Planar-optode pH and CO2 mapping for independent assessment of root-associated chemical gradients. |

# S8. Supplementary references

The following list provides complete bibliographic entries for the 51 author-verified paper-derived claim units in Table S4.

Aufrecht, J.A., Timm, C.M., Bible, A., Morrell-Falvey, J.L., Pelletier, D.A., Doktycz, M.J., et al., 2018. Quantifying the Spatiotemporal Dynamics of Plant Root Colonization by Beneficial Bacteria in a Microfluidic Habitat. Adv. Biosyst. 2, 1800048. https://doi.org/10.1002/adbi.201800048.

Bandara, C.D., Schmidt, M., Davoudpour, Y., Stryhanyuk, H., Richnow, H.H., Musat, N., 2021. Microbial Identification, High-Resolution Microscopy and Spectrometry of the Rhizosphere in Its Native Spatial Context. Front. Plant Sci. 12, 668929. https://doi.org/10.3389/fpls.2021.668929.

Bellgard, S.E., Padamsee, M., Probst, C.M., Lebel, T., Williams, S.E., 2016. Visualizing the early infection of Agathis australis by Phytophthora agathidicida, using microscopy and fluorescent in situ hybridization. Forest Pathol. 46, 622-631. https://doi.org/10.1111/efp.12280.

Blossfeld, S., Schreiber, C.M., Liebsch, G., Kuhn, A.J., Hinsinger, P., 2013. Quantitative imaging of rhizosphere pH and CO2 dynamics with planar optodes. Ann. Bot. 112, 267-276. https://doi.org/10.1093/aob/mct047.

Cao, Z., Zuo, W., Dai, L., 2023. Spatial profiling of microbial communities by sequential FISH with error-robust encoding. Nat. Commun. 14, 1477. https://doi.org/10.1038/s41467-023-37188-3.

Cao, Z.H., Zuo, W.L., Wang, L.X., Dai, L., 2026. Spatial mapping of microbial communities by an integrated automation platform of sequential FISH. Cell Reports Methods 6, 101381. https://doi.org/10.1016/j.crmeth.2026.101381.

Chen, B.D., Nayuki, K., Kuga, Y., Zhang, X., Wu, S.L., Ohtomo, R., 2018. Uptake and Intraradical Immobilization of Cadmium by Arbuscular Mycorrhizal Fungi as Revealed by a Stable Isotope Tracer and Synchrotron Radiation μX-Ray Fluorescence Analysis. Microbes Environ. 33, 257-263. https://doi.org/10.1264/jsme2.me18010.

Clode, P.L., Kilburn, M.R., Jones, D.L., Stockdale, E.A., Cliff, J.B., Herrmann, A.M., et al., 2009. In Situ Mapping of Nutrient Uptake in the Rhizosphere Using Nanoscale Secondary Ion Mass Spectrometry. Plant Physiol. 151, 1751-1757. https://doi.org/10.1104/pp.109.141499.

Downie, H., Holden, N., Otten, W., Spiers, A.J., Valentine, T.A., Dupuy, L.X., 2012. Transparent soil for imaging the rhizosphere. PLoS ONE 7, e44276. https://doi.org/10.1371/journal.pone.0044276.

Engelhardt, I.C., Holden, N., Daniell, T.J., Dupuy, L.X., 2024. Mobility and growth in confined spaces are important mechanisms for the establishment of Bacillus subtilis in the rhizosphere. Microbiology 170, 001477. https://doi.org/10.1099/mic.0.001477.

Fang, W., Williams, P.N., Zhang, H., Yang, Y., Yin, D.X., Liu, Z.D., et al., 2021. Combining Multiple High-Resolution In Situ Techniques to Understand Phosphorous Availability Around Rice Roots. Environ. Sci. Technol. 55, 13082-13092. https://doi.org/10.1021/acs.est.1c05358.

Fay, V., Zhang, O.M., Cao, R.Z., Yang, C.H.I., 2024. Improving planar optode lifetime: encapsulation and optical release of pH-sensing optodes and assessment of their lifetime in situ. J. Soils Sediments 24, 289-293. https://doi.org/10.1007/s11368-023-03650-4.

Fettrow, S., Rummel, P.S., James, W., Wilkins, M.J., Koren, K., Borch, T., 2026. From soil to gas - high resolution insights into plant-soil interactions by integrating planar oxygen optodes, porewater chemistry, soil microbial analysis and trace soil gas flux using a rhizobox approach. Soil Biol. Biochem. 218, 110149. https://doi.org/10.1016/j.soilbio.2026.110149.

Gamez, R.M., Ramirez, S., Montes, M., Cardinale, M., 2020. Complementary Dynamics of Banana Root Colonization by the Plant Growth-Promoting Rhizobacteria Bacillus amyloliquefaciens Bs006 and Pseudomonas palleroniana Ps006 at Spatial and Temporal Scales. Microb. Ecol. 80, 656-668. https://doi.org/10.1007/s00248-020-01571-0.

Ge, S.S., Dupuy, L.X., MacDonald, M.P., 2021. In situ laser manipulation of root tissues in transparent soil. Plant Soil 468, 475-489. https://doi.org/10.1007/s11104-021-05133-2.

Ge, Y.P., Ying, K.Y., Zhang, S.H., Wang, S.L., Fang, Y.Y., Huang, J., et al., 2025. Planar Optode Imaging Reveals Spatio-Temporal Heterogeneity of Rhizosphere Microecology in Celosia argentea Under Cadmium Stress. Toxics 14, 34. https://doi.org/10.3390/toxics14010034.

Gorka, S., Dietrich, M., Mayerhofer, W., Gabriel, R., Wiesenbauer, J., Martin, V., et al., 2019. Rapid Transfer of Plant Photosynthates to Soil Bacteria via Ectomycorrhizal Hyphae and Its Interaction With Nitrogen Availability. Front. Microbiol. 10, 168. https://doi.org/10.3389/fmicb.2019.00168.

Hlaváčková, K., Šamajová, O., Hrbáčková, M., Šamaj, J., Ovečka, M., 2023. Advanced microscopy resolves dynamic localization patterns of stress-induced mitogen-activated protein kinase (SIMK) during alfalfa root hair interactions with Ensifer meliloti. J. Exp. Bot. 74, 3729-3748. https://doi.org/10.1093/jxb/erad111.

Holz, M., Becker, J.N., Daudin, G., Oburger, E., 2020. Application of planar optodes to measure CO2 gradients in the rhizosphere of unsaturated soils. Rhizosphere 16, 100266. https://doi.org/10.1016/j.rhisph.2020.100266.

Honeker, L.K., Root, R.A., Chorover, J., Maier, R.M., 2016. Resolving colocalization of bacteria and metal(loid)s on plant root surfaces by combining fluorescence in situ hybridization (FISH) with multiple-energy micro-focused X-ray fluorescence (ME μXRF). J. Microbiol. Methods 131, 23-33. https://doi.org/10.1016/j.mimet.2016.09.018.

Jones, D.L., Clode, P.L., Kilburn, M.R., Stockdale, E.A., Murphy, D.V., 2013. Competition between plant and bacterial cells at the microscale regulates the dynamics of nitrogen acquisition in wheat (Triticum aestivum). New Phytol. 200, 796-807. https://doi.org/10.1111/nph.12405.

Koop-Jakobsen, K., Mueller, P., Meier, R.J., Liebsch, G., Jensen, K., 2018. Plant-Sediment Interactions in Salt Marshes - An Optode Imaging Study of O2, pH, and CO2 Gradients in the Rhizosphere. Front. Plant Sci. 9, 541. https://doi.org/10.3389/fpls.2018.00541.

Koop-Jakobsen, K., Wenzhöfer, F., 2015. The Dynamics of Plant-Mediated Sediment Oxygenation in Spartina anglica Rhizospheres-a Planar Optode Study. Estuaries Coasts 38, 951-963. https://doi.org/10.1007/s12237-014-9861-y.

Kreuzeder, A., Santner, J., Scharsching, V., Oburger, E., Hoefer, C., Hann, S., et al., 2018. In situ observation of localized, sub-mm scale changes of phosphorus biogeochemistry in the rhizosphere. Plant Soil 424, 573-589. https://doi.org/10.1007/s11104-017-3542-0.

Larsen, M., Santner, J., Oburger, E., Wenzel, W.W., Glud, R.N., 2015. O2 dynamics in the rhizosphere of young rice plants (Oryza sativa L.) as studied by planar optodes. Plant Soil 390, 279-292. https://doi.org/10.1007/s11104-015-2382-z.

Lenzewski, N., Mueller, P., Meier, R.J., Liebsch, G., Jensen, K., Koop-Jakobsen, K., 2018. Dynamics of oxygen and carbon dioxide in rhizospheres of Lobelia dortmanna - a planar optode study of belowground gas exchange between plants and sediment. New Phytol. 218, 131-141. https://doi.org/10.1111/nph.14973.

Li, X.Y., Li, S.Q., Jiang, Y.F., Yang, Q., Zhang, J.C., Kuzyakov, Y., et al., 2024. Multi-imaging platform for rhizosphere studies: Phosphorus and oxygen fluxes. J. Environ. Manage. 351, 119763. https://doi.org/10.1016/j.jenvman.2023.119763.

Liu, Y., Patko, D., Engelhardt, I., George, T.S., Stanley-Wall, N.R., Ladmiral, V., et al., 2021. Plant-environment microscopy tracks interactions of Bacillus subtilis with plant roots across the entire rhizosphere. Proc. Natl. Acad. Sci. U.S.A. 118, e2109176118. https://doi.org/10.1073/pnas.2109176118.

Liu, Y.M.H., Patko, D., de la Mata, A.L., Dong, X.S., Peral, E.G., He, X.H., et al., 2025. Microcosm fabrication platform for live microscopy of plant-soil systems. Biosyst. Eng. 252, 105-114. https://doi.org/10.1016/j.biosystemseng.2025.02.006.

Lohse, M., Haag, R., Lippold, E., Vetterlein, D., Reemtsma, T., Lechtenfeld, O.J., 2021. Direct Imaging of Plant Metabolites in the Rhizosphere Using Laser Desorption Ionization Ultra-High Resolution Mass Spectrometry. Front. Plant Sci. 12, 753812. https://doi.org/10.3389/fpls.2021.753812.

Lu, Y.H., Abraham, W.R., Conrad, R., 2007. Spatial variation of active microbiota in the rice rhizosphere revealed by in situ stable isotope probing of phospholipid fatty acids. Environ. Microbiol. 9, 474-481. https://doi.org/10.1111/j.1462-2920.2006.01164.x.

Ma, X.M., Mason-Jones, K., Liu, Y., Blagodatskaya, E., Kuzyakov, Y., Guber, A., et al., 2019. Coupling zymography with pH mapping reveals a shift in lupine phosphorus acquisition strategy driven by cluster roots. Soil Biol. Biochem. 135, 420-428. https://doi.org/10.1016/j.soilbio.2019.06.001.

Massalha, H., Korenblum, E., Malitsky, S., Shapiro, O.H., Aharoni, A., 2017. Live imaging of root-bacteria interactions in a microfluidics setup. Proc. Natl. Acad. Sci. U.S.A. 114, 4549-4554. https://doi.org/10.1073/pnas.1618584114.

Mayerhofer, W., Schintlmeister, A., Dietrich, M., Gorka, S., Wiesenbauer, J., Martin, V., et al., 2021. Recently photoassimilated carbon and fungus-delivered nitrogen are spatially correlated in the ectomycorrhizal tissue of Fagus sylvatica. New Phytol. 232, 2457-2474. https://doi.org/10.1111/nph.17591.

Meents, A.K., Furch, A.C.U., Almeida-Trapp, M., Özyürek, S., Scholz, S.S., Kirbis, A., et al., 2019. Beneficial and pathogenic Arabidopsis root-interacting fungi differently affect auxin levels and responsive genes during early infection. Front. Microbiol. 10, 380. https://doi.org/10.3389/fmicb.2019.00380.

Mittmann-Goetsch, J., Wilson, M., Jensen, K., Mueller, P., 2024. Root-Driven Soil Reduction in Wadden Sea Salt Marshes. Wetlands 44, 112. https://doi.org/10.1007/s13157-024-01867-8.

Noirot-Gros, M.F., Shinde, S.V., Akins, C., Johnson, J.L., Zerbs, S., Wilton, R., et al., 2020. Functional Imaging of Microbial Interactions With Tree Roots Using a Microfluidics Setup. Front. Plant Sci. 11, 408. https://doi.org/10.3389/fpls.2020.00408.

O'Callaghan, F.E., Braga, R.A., Neilson, R., MacFarlane, S.A., Dupuy, L.X., 2018. New live screening of plant-nematode interactions in the rhizosphere. Sci Rep 8, 1440. https://doi.org/10.1038/s41598-017-18797-7.

Parashar, A., Pandey, S., 2011. Plant-in-chip: Microfluidic system for studying root growth and pathogenic interactions in Arabidopsis. Appl. Phys. Lett. 98, 263703. https://doi.org/10.1063/1.3604788.

Patko, D., Yang, Q.Z., Liu, Y.M.H., Falireas, P., Briou, B., Tawade, B.V., et al., 2024. Smart soils track the formation of pH gradients across the rhizosphere. Plant Soil 500, 91-104. https://doi.org/10.1007/s11104-023-06151-y.

Quinn, L.K., Sharma, K., Faber, K.T., Orphan, V.J., 2025. Clear as mud redefined: Tunable transparent mineral scaffolds for visualizing microbial processes below ground. PNAS Nexus 4, pgaf118. https://doi.org/10.1093/pnasnexus/pgaf118.

Rodeghiero, M., Rubol, S., Bellin, A., Turco, E., Molinatto, G., Gianelle, D., et al., 2018. High Resolution Assessment of Spatio-Temporal Changes in O2 Concentration in Root-Pathogen Interaction. Front. Microbiol. 9, 1491. https://doi.org/10.3389/fmicb.2018.01491.

Rodionov, A., Bauke, S.L., von Sperber, C., Hoeschen, C., Kandeler, E., Kruse, J., et al., 2020. Biogeochemical cycling of phosphorus in subsoils of temperate forest ecosystems. Biogeochemistry 150, 313-328. https://doi.org/10.1007/s10533-020-00700-8.

Rudolph-Mohr, N., Bereswill, S., Tötzke, C., Kardjilov, N., Oswald, S.E., 2021. Neutron computed laminography yields 3D root system architecture and complements investigations of spatiotemporal rhizosphere patterns. Plant Soil 469, 489-501. https://doi.org/10.1007/s11104-021-05120-7.

Schmidt, H., Gorka, S., Seki, D., Schintlmeister, A., Woebken, D., 2023. Gold-FISH enables targeted NanoSIMS analysis of plant-associated bacteria. New Phytol. 240, 439-451. https://doi.org/10.1111/nph.19112.

Scholz, V.V., Müller, H., Koren, K., Nielsen, L.P., Meckenstock, R.U., 2019. The rhizosphere of aquatic plants is a habitat for cable bacteria. FEMS Microbiol. Ecol. 95, fiz062. https://doi.org/10.1093/femsec/fiz062.

Spohn, M., Treichel, N.S., Cormann, M., Schloter, M., Fischer, D., 2015. Distribution of phosphatase activity and various bacterial phyla in the rhizosphere of Hordeum vulgare L. depending on P availability. Soil Biol. Biochem. 89, 44-51. https://doi.org/10.1016/j.soilbio.2015.06.018.

Strömberg, N., 2008. Determination of ammonium turnover and flow patterns close to roots using Imaging optodes. Environ. Sci. Technol. 42, 1630-1637. https://doi.org/10.1021/es071400q.

Vidal, A., Hirte, J., Bender, S.F., Mayer, J., Gattinger, A., Höschen, C., et al., 2018. Linking 3D Soil Structure and Plant-Microbe-Soil Carbon Transfer in the Rhizosphere. Front. Environ. Sci. 6, 9. https://doi.org/10.3389/fenvs.2018.00009.

Walton, C.L., Khalid, M., Bible, A.N., Kertesz, V., Retterer, S.T., Morrell-Falvey, J., et al., 2022. In Situ Detection of Amino Acids from Bacterial Biofilms and Plant Root Exudates by Liquid Microjunction Surface-Sampling Probe Mass Spectrometry. J. Am. Soc. Mass Spectrom. 33, 1615-1625. https://doi.org/10.1021/jasms.2c00081.

Watt, M., Hugenholtz, P., White, R., Vinall, K., 2006. Numbers and locations of native bacteria on field-grown wheat roots quantified by fluorescence in situ hybridization (FISH). Environ. Microbiol. 8, 871-884. https://doi.org/10.1111/j.1462-2920.2005.00973.x.
